# Efficacy analysis and research progress of complementary and alternative medicines in the adjuvant treatment of COVID-19

**DOI:** 10.1186/s12929-023-00923-5

**Published:** 2023-05-03

**Authors:** Jaung-Geng Lin, Guan-Jhong Huang, Yi-Chang Su

**Affiliations:** 1 School of Chinese Medicine, College of Chinese Medicine, China Medical University, No.91, Hsueh-Shih Road, Taichung, 40402 Taiwan; 2 Chinese Medicine Research Center, China Medical University, No.91, Hsueh-Shih Road, Taichung, 40402 Taiwan; 3 Department of Chinese Pharmaceutical Sciences and Chinese Medicine Resources, College of Chinese Medicine, China Medical University, No.91, Hsueh-Shih Road, Taichung, 40402 Taiwan; 4 Department of Food Nutrition and Healthy Biotechnology, Asia University, No. 500, Lioufeng Road, Taichung, 41354 Taiwan; 5 National Research Institute of Chinese Medicine, Ministry of Health and Welfare, No.155-1, Section 2, Linong Street, Beitou District, Taipei, 11221 Taiwan

**Keywords:** SARS-CoV-2, Complementary and alternative medicines, Viral infection, Antivirus, Anti-inflammation, NRICM102

## Abstract

The coronavirus disease 2019 (COVID-19) pandemic has impacted human lifestyles around the world, causing huge distress in terms of public health systems, emergency response capacity and economic development. The causative agent of COVID-19, severe acute respiratory syndrome coronavirus 2 (SARS-CoV-2), is associated with respiratory involvement, cardiovascular-related diseases, and ultimately causes multiple organ failure and death in severely affected individuals. Thus, effective prevention or early treatment of COVID-19 is critical. An effective vaccine offers a way out of the pandemic for governments, the scientific community and people worldwide, but we still lack effective drug therapies, including treatments for the prevention and treatment of COVID-19. This had led to a high global demand for many complementary and alternative medicines (CAMs). Moreover, many healthcare providers are now requesting information about CAMs that prevent, relieve, or treat the symptoms of COVID-19 and even alleviate vaccine-related side effects. Experts and scholars must therefore become familiar with the use of CAMs in COVID-19, current research directions and effectiveness of CAMs for COVID-19. This narrative review updates the current status and research worldwide on the use of CAMs for COVID-19. The review provides reliable evidence on theoretical viewpoints and therapeutic efficacies of CAM combinations, and evidence in support of the therapeutic strategy of Taiwan Chingguan Erhau (NRICM102) against moderate-to-severe novel coronavirus infectious disease in Taiwan.

## Introduction

The COVID-19 pandemic, which spread rapidly around the world after emerging in Wuhan, China in December 2019, remains a global health problem [1], with more than 750 million cases and 6.8 million deaths related to COVID-19 reported by March 2023, with individuals who have compromised immune systems or chronic diseases and the elderly being at high risk (https://covid19.who.int/). Despite World Health Organization (WHO) prevention guidelines intended to slow the spread of the virus, the COVID-19 pandemic continues to greatly affect human physiology and psychology. Inadequate prevention and treatment measures, as well as the rapid spread of COVID-19 infection contribute to the perceived unknown risks to humans, so many protective and preventive behaviors have emerged as attempts to reduce these disease-related risks [2, 3]. In addition to the conventional interventions, such as emergency-authorized vaccines and antiviral drugs that are designed to reduce risk and better manage processes to prevent or treat COVID-19 infections, complementary or alternative treatments such as herbal preparations, dietary therapies and vitamin supplements can be used [4–6].

CAMs, including traditional herbal remedies, are being implemented in many countries as an option to prevent or treat COVID-19 infections. During the COVID-19 pandemic, it is very important to enhance personal immunity, reduce the side effects of antiviral or anti-inflammatory drugs, and protect individuals from this disease [3, 7]. CAMs are widely believed to improve the quality of life, attitudes towards self-care, and an individual’s health status, although there is currently only limited evidence to support the usefulness of CAMs in reducing viral load and/or symptoms. Some countries, including Taiwan, China and India have engaged in pilot studies designed to assess the efficacies of traditional medicines against coronavirus infections. Traditional Chinese medicine (TCM) has been used for many centuries to treat infectious diseases according to their clinical features and symptoms [8, 9]. Much study evidence claims that TCM has antiviral properties that can improve recovery rates, shorten disease duration, and reduce mortality. The ease of use of TCM and its low cost have contributed to its increased use [10–12].

A bedside-to-bench analysis of clinical outcomes and pharmacology has highlighted the therapeutic efficacy of TCM for improving symptoms of pulmonary infiltrates of COVID-19 [13]. The Department of Traditional Chinese Medicine in Taiwan’s Ministry of Health and Welfare has issued guidelines for the clinical staging of treatment of COVID-19 virus [14]. TCM therapies offer promise for the development of COVID-19 drugs in Taiwan [15].

### Small molecule therapies for COVID-19

The genetic diversification of SARS-CoV-2 is worryingly rapid, with multiple variants emerging by late 2020 that exacerbated regional epidemics; all five variants of concern (VOC) identified so far (Alpha, Beta, Gamma, Delta, and Omicron) have mutations that encourage their evasion of host immune responses [16]. The spike glycoprotein appears to be particularly prone to accumulating mutations. A vaccine based on the spike protein has been developed and the vaccination program is being given maximum global support. However, serum studies and evidence suggest that Omicron escapes immunity, regardless of prior infection or vaccination [17].

Direct-acting antiviral drugs against SARS-CoV-2 are classified as either monoclonal antibodies (mAbs) against the spike protein, or small molecules that interfere with viral replication. The SARS-CoV-2 VOC Omicron is insensitive to most currently approved mAbs, which have shown poor clinical efficacy [18]. Currently available direct-acting small-molecule SARS-CoV-2 antivirals target the conserved viral RNA-dependent RNA polymerase (RdRp), the conserved viral major protease (the 3C-like protease [3CL^pro^]), or the main protease (M^pro^). Small-molecule therapeutics remdesivir, molnupiravir and Paxlovid (nirmatrelvir plus ritonavir) have shown significant efficacy in the treatment of COVID-19 and been therefore been granted emergency use authorization or have entered phase III trials in many countries [19]. Moreover, many other anti-SARS-CoV-2 drug candidates have been developed and have now entered clinical evaluation. It is hoped that these new drugs will overcome COVID-19.

Remdesivir, originally used to treat the Ebola virus, inhibits the RdRp of SARS-CoV-2. It is the first antiviral to be approved or authorized for emergency use to treat COVID-19, with evidence showing that remdesivir improves the clinical outcomes of hospitalized patients with moderate-to-severe disease and prevents the progression of COVID-19 in outpatients [20].

The oral biologic molnupiravir also inhibits viral RdRp and was originally used against different RNA viruses such as influenza. In a phase 2a clinical trial, molnupiravir accelerated the clearance of SARS-CoV-2 RNA and eliminated infectious virus in patients with COVID-19 [21]. In the UK, molnupiravir has been authorized for use in patients with mild-to-moderate COVID-19 and at least one risk factor for developing severe disease [22]. The UK-based phase 2 trial (AGILE CST-2) involving vaccinated and unvaccinated patients with early COVID-19 infection showed a moderate antiviral effect of molnupiravir [23]. In the UK-based PANORAMIC trial involving more than 25,000 participants, the addition of molnupiravir to usual care for vaccinated adults in the community with confirmed COVID-19 and at increased risk of adverse outcomes was associated with reduced time to recovery overall and for key individual symptoms, reduced healthcare seeking for some primary care services, and reduced viral load [24]. The US Food and Drugs Administration (FDA) granted an Emergency Use Authorization (EUA) in 2021 for molnupiravir in adults with mild-to-moderate COVID-19 who are at high risk of developing a severe infection and who either cannot access or are deemed clinically inappropriate for alternative COVID-19 treatment options [25].

The oral antiviral paxlovid consists of nirmatrelvir (an irreversible inhibitor of the SARS-CoV-2 M^pro^) and ritonavir. About 90% of people are protected from severe COVID-19 and hospitalization if paxlovid treatment starts in the first few days after symptoms appear. Interestingly, evolutionary mutations can occur that affect the M^pro^ gene, but do not appear to alter the antiviral potency of nirmatrelvir [26].

While these antivirals are expected to reduce hospitalizations and deaths among patients with COVID-19, new virus variants continue to emerge with mutations that may provide SARS-CoV-2 with the ability to evade immunity, fueling future waves of infection and diminishing treatment options. The world urgently needs therapeutic strategies capable of attacking SARS-CoV-2 from multiple aspects.

### The global development of CAMs against COVID-19

Due to the various challenges posed by COVID-19, governments, healthcare professionals and pharmaceutical companies are investing enormous efforts to control the disease worldwide. The scientific community is experimenting with new interventions [27]. Countries with a long history of traditional medicine such as China, India and Taiwan are exploring the effectiveness of their traditional medicines in the treatment of COVID-19.

### China

TCM is recommended in China by the National Health Commission (NHC) guidelines for the treatment of COVID-19 [9]. The TCM concept of personalized treatment through syndrome differentiation enables clinicians to summarize pathological bodily signs during the disease course, based on diagnostic data collected from patients via observation, listening and smelling, asking, palpation and pulse‐taking [28].^1^

Up until February 2023, 15 TCMs are recommended in China for the treatment of COVID-19: seven oral formulations (Angong Niuhuang, Zixue, Huoxiang Zhengqi, Jinhua Qinggan, Lianhua Qingwen, Shufeng Jiedu, and Fangfeng Tongsheng) and eight injectable formulations (Xiyanping, Xuebijing, Reduning, Tanreqing, Xingnaojing, Shenfu, Shengmai, and Shenmai). The China Food and Drug Administration (CFDA) has approved Xuebijing for treating new coronavirus pneumonia with severe and critical systemic inflammatory response syndrome or/and multiple organ failure, and Lianhua Qingwen for treating fever, cough, and fatigue caused by the light and ordinary types of the new coronavirus pneumonia [29, 30]; each TCM formulation consists of several active ingredients with multitarget effects that are intended to counteract drug resistance. In particular, two TCM formulations (the Reyanning mixture and Jingyin granules) have shown potent anti-inflammatory and immunomodulatory effects [31, 32]. China is now conducting many clinical trials assessing the effectiveness of various TCM formulations for the treatment of COVID-19.

### India

In view of the contagiousness and severity of COVID-19 infection, India’s Ministry of AYUSH (Ayurveda, Yoga and Naturopathy, Unani, Siddha and Homeopathy) recommends a decoction of ginger, curcumin, cloves, honey, fennel seeds, cumin seeds in warm water as an immune booster [33]. Nonpharmacologic Ayurvedic preventive measures include a healthy lifestyle, adequate physical activity, adequate sleep, and avoidance of patients with COVID-19. Ayurvedic medicines including garlic (*Allium sativum* L.), turmeric (*Curcuma longa* L.), carom or ajwain (*Trachyspermum ammi* L.) can be used to prevent COVID-19 [34]. Numerous studies in India have reported antiviral properties of Ayurvedic preparations in COVID-19 treatment. *In vitro* evidence shows that *Withania somnifera* (Ashwagandha), *Tinospora cordifolia* (Giloy) and *Ocimum sanctum* (Tulsi) demonstrate protease inhibitory activity against SARS-CoV-2 [35]. The WHO supports *Artemisia annua* (abundant in India and China) as a drug research candidate for treating COVID-19 [36, 37].

### Taiwan

The Department of TCM of the Ministry of Health and Welfare of Taiwan encourages TCM clinical services for treating COVID-19 [38]. The Chinese medicine formulations Jing Si Herbal Tea, Jing Guan Fang, Taiwan Chingguan Yihau (NRICM101) and NRICM102 that have been launched since 2020 have all been developed and applied in Taiwan and show promise in the treatment of COVID-19 (Table 1).

**Table 1:** Possible therapeutic effects of the Chinese medicine formulations in COVID-19 treatment

Formula	Composition (% of total weight)	Participants performance	Pharmacological effects	References
Jing Si Herbal Tea	*Artemisia argyi* (21.28%) *Anisomeles indica* (21.28%) *Houttuynia cordata* (14.18%) *Platycodon grandifloras* (14.18%) *Ophiopogon japonicus* (14.18%) *Perilla frutescens* (7.09%) *Glycyrrhiza glabra* (7.09%) *Chrysanthemum morifolium* (0.71%)	Patients (n = 117) had lower risks of intubation, Medisave Care Unit admission, and mortality	Antiviral, anti-inflammatory, antioxidative, antithrombotic activity	[40]
Jing Guan Fang	*Forsythia suspensa* (30.3%) *Scutellaria baicalensis* (24.2%) *Bupleurum Chinese* (18.2%) *Magnolia officinalis* (18.2%) *Agastache rugose* (9.0%)	Patients (n = 151) who received NRICM101 plus usual care did not reach the study endpoint during the 30-day observation period	–	[41]
NRICM101	*Scutellaria baicalensis* (13.88%) *Houttuynia cordata* (13.88%) *Trichosanthes kirilowii* (13.88%) *Isatis indigotica* (13.88%) *Morus alba* (8.33%) *Magnolia officinalis* (8.33%) *Mentha haplocalyx* (8.33%) *Nepeta tenuifolia* (8.33%) *Saposhnikovia divaricate* (5.55%) *Glycyrrhiza glabra* (5.55%)	Patients (n = 12) achieved 3 consecutive negative results within a median of 9 days and reported no adverse events	Inhibited the spike protein/ACE2 interaction, 3CL protease activity; antiviral, anti-inflammatory activity	[13]
NRICM102	*Houttuynia cordata* (23.25%) *Scutellaria baicalensis* (11.62%) *Trichosanthes kirilowii* (11.62%) *Artemisia scoparia* (11.62%) *Wolfiporia extensa* (11.62%) *Magnolia officinalis* (6.97%) *Polygonatum odoratum* (6.97%) *Pinellia ternata* (6.97%) *Glycyrrhiza glabra* (4.65%) *Aconitum carmichaelii* (4.65%)	Patients (n = 123) who received NRICM102 plus usual care were 74.07% less likely to die than non-users	Disrupted spike protein/ACE2 interaction, 3CL protease activity; anti-inflammatory activity; regulated TLRs, JAK/STAT, PI3K/AKT, and NET signaling pathways	[15]
			–	

### Jing Si herbal drink

Jing Si herbal drink (JSHD), a formula jointly developed by Tzu Chi University and Tzu Chi Hospital in Taiwan, contains a total of eight medicinal materials. The ingredients of JSHD include *Artemisia argyi* (21.28%), *Anisomeles indica* (21.28%), *Houttuynia cordata* (14.18%), *Platycodon grandifloras* (14.18%), *Ophiopogon japonicus* (14.18%), *Perilla frutescens* (7.09%), *Glycyrrhiza glabra* (7.09%), *Chrysanthemum morifolium* (0.71%) **(**Table 1**)**. In the treatment of COVID-19 infection, JSHD shows good antiviral activity (blocking angiotensin converting enzyme-2 [ACE2], transmembrane protease, serine 2 [TMPRSS2] and 3C-like protease [3CL^pro^] activity), antioxidant activity (increasing superoxide dismutase (SOD) activity), anti-inflammatory activity (decreasing inducible nitric oxide synthase [iNOS] expression and nuclear factor-κB [NF-κB] phosphorylation, inhibiting nitric oxide [NO], tumor necrosis factor-α [TNF-α] and interleukin 6 [IL-6] production), suppression of immune system overactivity (reducing macrophages, neutrophils, and leukocytes), and attenuation of organ damage [39]. JSHD shows potential for the inhibition and treatment of COVID-19, although further studies are needed to evaluate possible interactions between JSHD and Chinese herbal components in different pathways, as well as the effects of different drugs on the pharmacologic activities of JSHD. For now, this formulation is deemed appropriate as an adjunctive agent in the COVID-19 treatment armamentarium. More clinical evidence is awaited as to the efficacy and safety of JSHD in COVID-19 infection [40].

### Jing Guan Fang

Jing Guan Fang (JGF) is the product of a research team led by Dr. Xu Zhonghua from the Yangming Jiaotong University Institute of Traditional Medicine and Taipei City Hospital. This research team designed JGF with reference to TCM theory described in the ancient medical classic “Wen Epidemic”. JGF consists of five herbs: *Forsythia suspensa* (Thunb.) Vahl (monarch), *Scutellaria baicalensis* (minister), *Bupleurum Chinese* (adjuvant), *Magnolia officinalis* Rehder and E.H. Wilson (adjuvant) and *Agastache rugose* (Fisch. and C.A. Mey.) Kuntze (courier) [41].

JGF inhibits syncytia and viral plaque formation and has not shown toxicity in cellular and preclinical studies [41]. JGF has induced the degradation of lysosome-dependent ACE2 and suppressed levels of TMPRSS2 mRNA and protein in human lung WI-38 and MRC-5 cells, and lowered protein levels of ACE2 and TMPRSS2 in lung tissues of mice after inhaling JGF [41]. These findings suggest that JGF has potential as an adjunctive preventive strategy against SARS-CoV-2 infection [41].

#### Taiwan Chingguan Yihau

NRICM101 was developed in 2020 by Taiwan’s National Research Institute of Chinese Medicine (NRICM) for alleviating mild and moderate cases of infectious pneumonia. The dosage of NRICM101 is increased or decreased according to “mild decoction pieces” (for details, refer to the NRICM instruction manual: https://www.nricm.edu.tw/p/406-1000-6500,r61.php?Lang=en). NRICM101 originates from the “Jing Fang Baidu Powder” TCM formula detailed in “The Prescriptions for Taking Lives” compiled by Zhang Shiche during the Ming Dynasty. The original recipe contains *Nepeta*, Fangfeng, bupleurum, poria, platycodon, *Chuan Xiong*, Qiang Huo, Duhuo, *Citrus aurantium*, licorice, ginger, and other medicinal materials. As the main clinical manifestations of patients with novel coronavirus disease consist of “disease entering the lung and transforming heat”, the prescription has been adjusted to use “Nepeta, Fangfeng, Mint, Mulberry Leaf” as the monarch medicine, “*Scutellaria baicalensis, Radix isatidis, Houttuynia cordata*” used as ministerial medicines, “Quan Gulou” for chest and an expectorant and “Hopu” for lowering qi and relieving asthma are used as adjuvant medicines, while “Licorice” is used as agent to reconcile the entire recipe [13, 14]. Thus, the ingredients of NRICM101 are mint, nepeta, mulberry leaf, parsnip, skullcap, *Houttuynia cordata*, northern isatis root, cogongrass, magnolia bark, and licorice.

At the time of writing this review, 14 GMP Chinese medicine factories have obtained authorization for the manufacture of NRICM101; 12 products prepared by these GMP factories have been granted a special drug license for export and a temporary drug license (EUA) by Taiwan’s Ministry of Health and Welfare, and the rights to produce and export to almost 60 countries worldwide that have experienced severe COVID-19 epidemics (including Europe, America, Asia, Africa and Australia) [42]. NRICM101 has successively obtained the following import rights: the “Singapore Drug Certificate”, “Australian Drug Certificate”, “Thailand Herbal Medicine License”, “EU Supplementary Food Registration Certificate”, “Philippine Herbal Medicine Granule Registration”, “Cambodia Dietary Supplement Registration”, “UK Import Permit”, “US Import Permit”, “Canada Import Permit”, and the “South Africa Import Permit”.

Studies of NRICM101 have demonstrated multiple targeting mechanisms, including the blockade of the COVID-19 spike protein receptor-binding domain by binding to the host cell's ACE2 receptor and inhibiting viral infection, inhibiting 3CL^pro^ activity and blocking host cell viral replication, inhibiting alveolar macrophages that secrete proinflammatory cytokines (TNF-α and IL-6) and thus stopping the formation of cytokine storms, reducing lung injury and the development of pulmonary fibrosis. The experience with NRICM101 in Taiwan suggests that this multitargeted drug candidate has good safety and therapeutic efficacy in COVID-19 [13, 14].

#### Taiwan Chingguan Erhau

NRICM102 was developed by Taiwan’s NRICM in 2021 and has been clinically tested for the adjuvant treatment of severely and critically ill patients with COVID-19. NRICM102 is derived from NRICM101 by adding or subtracting five herbs (Table 2) [13]. The antiviral and immune-regulating drugs remain unchanged, while the drugs for enhancing and repairing human tissue have been added. While NRICM101 is formulated for patients with mild and moderate new coronary pneumonia who are classified as below WHO grade 4 (no oxygen treatment required), NRICM102 is intended for severe-to-critically ill patients with new coronary pneumonia and a WHO clinical classification of grade 5 or above (oxygen therapy is required) [14, 15].

**Table 2:** Comparison of the medicinal ingredients in NRICM101 and NRICM102 [13, 15]

Chinese name/Latin name	NRICM101 (g)	NRICM102 (g)
Sang Ye* (Morus alba)*	11.25	-
Fangfeng* (Saposhnikovia divaricata)*	7.50	-
Banlangen* (Isatis tinctoria)*	18.75	-
Bohe* (Mentha canadensis)*	11.25	-
Jingjie* (Nepeta tenuifolia)*	11.25	-
Huangqin *(Scutellaria baicalensis)*	18.75	18.75
Yuxingcao *(Houttuynia cordata)*	18.75	37.50
Gancao* (Glycyrrhiza uralensis)*	7.50	7.50
Houpo *(Magnolia officinalis)*	11.25	11.25
Gualou* (Trichosanthes kirilowii)*	18.75	18.75
Yinchen* (Artemisia scoparia)*	-	18.75
Fuling *(Wolfiporia extensa)*	-	18.75
Yuzhu *(Polygonatum odoratum)*	-	11.25
Pinellia *(Pinellia ternata)*	-	11.25
Fuzi *(Aconitum carmichaelii)*	-	7.50

The formula of NRICM102 is composed of 10 herbs, including *Scutellaria baicalensis*, *Houttuynia cordata*, Trichosanthes, Pao Fuzi, *Magnolia officinalis*, poria, Polygonatum, *Pinellia sinensis*, Mian Yin Chen and Zhi Gan Cao (Table 3). NRICM102 has proven effective in improving pulmonary fibrosis in animal experiments, and good results have also been found in preliminary clinical trials exploring the same therapeutic indication [14, 15]. Studies have shown that NRICM102 has five major effects: inhibiting the activity of SARS-CoV-2 virus; immunomodulatory activity—inhibiting spike protein-induced cytokine expression activity; inhibiting acute lung inflammation injury activity; pulmonary embolism screening protection activity; prevention and improvement of lung fibrotic potential.

**Table 3:** Overview of NRICM101 and NRICM102 ingredients and the therapeutic efficacies of each ingredient

Chinese name	English/ Latin name	Family	Genus	Prescription functions
Sang Ye	Mulberry Leaf*/ Morus alba* L	Moraceae Gaudich	Morus Linn	Clears heat, moistens the lungs and relieves cough
Fangfeng	Siler*/ Saposhnikovia divaricata* (Turcz. ex Ledeb.) Schischk	Apiaceae	Saposhnikovia	Dispel wind and heat, eliminate muscle pain, reduce fever
Banlangen	Radix isatidis/ *Isatis tinctoria* subsp. tinctoria	Cruciferous	Isatis	Antibacterial and antiviral effects
Bohe	Mint/ *Mentha canadensis* L	Lamiaceae	Mentha	Cough and phlegm, heat, relieve muscle pain
Jingjie	Fineleaf Schizonepeta Herb/ *Nepeta tenuifolia* Benth. / *Schizonepeta tenuifolia*	Lamiaceae	Schizonepeta	Evacuate wind and heat, eliminate muscle soreness
Huangqin	Scutellaria/*Scutellaria baicalensis* Georgi	Lamiaceae	Scutellaria	Clears Shangjiao wind-heat, antibacterial and antiviral activities
Yuxingcao	Fishwort/*Houttuynia cordata* Thunb	Saururaceae	Houttuynia	Bactericidal antiviral cough
Fuzi	Prepared Monkshood Daughter Root*/Aconitum carmichaeli* Debeaux	Ranunculaceae	Aconitum	Warms the Yang, disperses cold and relieving pain
Fuling	Indian Buead/*Wolfiporia extensa* (Peck) Ginns	Polyporaceae	Wolfiporia	Dispels dampness, strengthens the spleen and calms the heart
Gualou	Trichosanthes root/*Trichosanthes kirilowii* Maxim	Cucurbitaceae	Trichosanthes	Removes hot phlegm and clears lung heat to improve inflammation of the lungs
Yuzhu	Fragrant Solomonseal Rhizome/*Polygonatum odoratum* (Mill.) Druce	Asparagaceae; Liliaceae	Polygonatum	Nourishes the Yin of the lungs, generates body fluid and quenches thirst
Gancao	Licorice*/Glycyrrhiza uralensis* Fisch. ex DC	Leguminosae	Glycyrrhiza	Expels phlegm and relieves cough, clears heat and detoxifies, protects the stomach and intestines
Houpo	Officinal Magnolia Bark/*Magnolia officinalis* Rehder & E.H. Wilson	Magnoliaceae	Magnolia	Can widen and calm the breath
Yinchen	Capillary Wormwood Herb/*Artemisia scoparia* Waldst. et Kit	Asteraceae; Compositae	Artemisia	Clears dampness and heat, normalizes bile and cures jaundice
Pinellia	Ternate Pinellia; Pinellia tuber/*Pinellia ternata* (Thunb.) Makino	Araceae	Pinellia	Dries dampness and resolves phlegm, calms the upward perverted flow of Qi and arrests vomiting

### Therapeutic efficacy of NRICM101 and NRICM102 in COVID-19 treatment

Data from studies and mechanisms of action for all NRICM101 and NRICM102 compounds and their active constituents are described in detail below and summarized in Table 4.

1. Mulberry leaf (*Morus alba* L.)
  Mulberry leaves are one of the most commonly used Chinese herbal medicines and are an excellent source of functional nutraceuticals. The traditional usage is to treat wind-cold headache, dizziness, cough, bronchitis and asthma. Mulberry leaf has many biological activities such as antioxidant, antibacterial, anti-inflammatory, hypoglycemic, and anti-aging [43]. Kuwanon X from *M. alba* leaf have shown anti-infective activities, particularly against herpes simplex virus 1 (HSV-1), herpes simplex virus 2 (HSV-2) replication and inhibited the HSV-1-induced nuclear factor (NF)-κB activation [44]. M. alba aqueous extract has exhibited activity against influenza viruses (H1N1 and H3N2) [45]. Notably, M. alba extract was found to block SARS-CoV-2 cell entry by inhibiting biological processes required for TMPRSS2 using molecular dynamics simulations [46]. Mulberry leaf flavonoids inhibited the production of inflammatory cytokines and decreased expression of NO, iNOS, cyclooxygenase-2 (COX-2) in lipopolysaccharide (LPS)-stimulated RAW264.7 cells [47].
2. Saposhnikovia root (*Saposhnikovia divaricata* (Turcz. ex Ledeb.) Schischk.)
  *S. divaricata* is a perennial herb native to northern Asia. Traditional Chinese medicine is used for dizziness, headache, and body aches. *S. divaricata* has been found to have inhibitory effects on peptic ulcer, reduce fever, and possess analgesic, antioxidant and anti-inflammatory activities [48]. Some evidence suggests that three new coumarins from *S. divaricata* inhibits porcine epidemic diarrhea virus (PEDV) activity [49]. Ethanol extracts of *S. divaricata* showed antiinflammatory and antiosteoarthritis effects reduced the LPS-induced NO, prostaglandin E2 (PGE2), TNF-α, and IL-6 in RAW 264.7 cells [50].
3. Indigowoad root (*Isatis tinctoria* subsp. tinctoria)
  During the SARS outbreak in China, Hong Kong, and Taiwan, *I. indigotica* were often used to prevent SARS. *I. indigotica* root has been found to have antiviral effects against influenza, hepatitis A, and Japanese encephalitis. *I. Indigo* root contains indigo, indirubin, indigo (indoxyl-β-d-glucoside), β-sitosterol, γ-sitosterol and sinigrin [51]. Indigo, sinigrin, aloe-emodin, and hesperetin were found in *I. indigotica* roots to show anti-influenza virus effects by blocking 3CL^pro^ cleavage [52]. Lariciresinol from *I. indigotica* inhibits hepatitis B virus by regulating viral transcription [53]. The petroleum ether extract of *I. indigotica* showed anti-inflammatory properties by downregulating inflammatory cytokines such as IL-6 and interleukin-33, inhibiting mast cell responses, and COX-2 activity in a murine model [54].
4. Peppermint herb (*Mentha canadensis* L.)
  *M. canadensis* is widely used in food, cosmetics and pharmaceuticals. In Chinese medicine, M. canadensis is used to treat diseases related to the nervous, respiratory, reproductive and digestive systems. Pharmacological studies of *M. canadensis* revealed antibacterial, anti-inflammatory, antiviral, antioxidant, antitumor, antidiabetic, cardioprotective and hepatoprotective activities, mainly due to its antioxidant potential as well as low toxicity and high efficacy results [55]. *M. canadensis* contains many volatile compounds (such as menthol and menthone) as well as polyphenolic acids, flavonoids and monoterpenoids [55]. Furthermore, *M. canadensis* effectively prevents disease development in a SARS-CoV-2-infected hamster disease model [56]. *M. arvensis* essential oil exerts anti-inflammatory in LPS-stimulated inflammatory responses via inhibiting inflammatory cytokines and extracellular signal regulated kinase (ERK)/NF-κB signaling pathway and anti-atopic dermatitis-like effects in 2,4-dinitrochlorobezene-induced mice [57].
5. *Nepeta tenuifolia* (*Schizonepeta tenuifolia* Briq.)
  *N. tenuifolia* has a long history of medicinal use in China, Taiwan, Japan, and Korea. It is traditionally used in the treatment of fever, headache, viral infections, and sore throat, and has anti-inflammatory, immunomodulatory, antioxidant, and antipruritic activities [58]. The main chemical constituents of N. tenuifolia are volatile oils, with isomenthone and menthone being the two most abundant constituents [58]. *S. baicalensis* extracts inhibited virus replication through the induction of antiviral interferon production [59, 60]. The anti-inflammatory effects of an aqueous extract of *N. tenuifolia* on LPS-induced TNF-α and IL-6 in vivo and in vitro [61].
6. Scutellaria root (*Scutellaria baicalensis* Georgi)
  For TCM purposes, *S. baicalensis* is used to clear heat, purge fire, detoxify, and achieve hemostasis. *S. baicalensis* has shown antitumour, antiviral, antimicrobial, and anti-inflammatory activities [62]. *S. baicalensis* extracts are active against Zika, influenza A (H1N1), human immunodeficiency virus (HIV) and dengue virus (DENV) viruses. Flavonoid ingredients (baicalin, baicalein and wogonin) in *S. baicalensis* inhibit the in *vitro* replication of SARS-CoV-2 and 3CL^pro^ [62, 63], and significantly lower NO production and levels of TNF-α, IL-6, and monocyte chemoattractant protein-1 (MCP-1) expression [64]. *S. baicalensis* water extract significantly inhibits NO production, interleukins 3, 6, 10, 12p40 and 17, interferon-inducible protein-10 (CXCL-10), vascular endothelial growth factor (VEGF) and keratinocyte-derived chemokines in LPS-stimulated RAW 264.7 macrophages [65].
7. Yuxingcao (*Houttuynia cordata* Thunb.)
  *H. cordata* has traditionally been used to treat pneumonia, bronchitis, and chronic obstructive respiratory disease. Scientific analysis has identified antiviral, anti-inflammatory, and antioxidative activities in *H. cordata* extract [66]. *H. cordata* is also active against herpes simplex virus 1 (HSV-1), influenza virus, and HIV type 1 (HIV-1) [67, 68].
  *H. cordata* essential oil inhibits COX-2, while *H. cordata* aqueous extracts reduce influenza symptoms and LPS-induced lung injury [69]. The *H. cordata* flavonoid quercitrin shows anti-inflammatory activity [70] and reduces cell numbers in the bronchoalveolar fluid of mice with LPS-induced lung injury [71]. Quercitrin also helps to prevent body weight loss, reductions in viral titers and mortality in mice infected with influenza A/WS/33 virus [72].
8. Gancao (*Glycyrrhiza uralensis* L*.*)
  The most important medicinal parts of *G. glabra* L. (Fabaceae family), commonly known as licorice, are the rhizomes and roots, which are used alone or in combination with other herbs to treat digestive system disorders (e.g., stomach ulcers, hyperdipsia, flatulence, colic), respiratory tract disorders (e.g., asthma, coughs, sore throat, and tonsillitis), jaundice, hemorrhagic diseases, malaria, fever, epilepsy, sexual dysfunction, paralysis, rheumatism, leucorrhea, psoriasis, and prostate cancer. Licorice is used as a food and beverage flavoring [73].
  The active compounds in *G. glabra* roots include the flavonoids glucoliquiritin, licoarylcoumarin, licopyranocoumarin, liquirtin, liquiritigenin, isoliquiritigenin, rhamnoliquirilin, and glycyrrhizin. Notably, glycyrrhizin can inhibit virus-cell binding and has proven efficacy in HIV-1 and chronic hepatitis C virus (HCV) infections [74]. Glycyrrhizin markedly inhibits SARS viral reproduction [75, 76]. Both glycyrrhizin and isoliquiritigenin effectively suppress LPS-induced activation of signaling cascades and cytokine production [77].
9. Houpo (*Magnolia officinalis* Rehder & E.H. Wilson)
  *M. officinalis* contains several biologically active compounds: 4-O-methylhonokiol, honokiol, magnolol, obovatol, and other neolignan compounds. In particular, honokiol and magnolol exhibit antitumorigenic, anti-inflammatory, antithrombotic, antioxidative, and neuroprotective activities [78]. *M. officinalis* extract suppresses human norovirus surrogates, murine norovirus (MNV) and feline calicivirus (FCV) in vitro and reduces MNV and FCV titers to undetectable levels in model food systems [79]. Honokiol can also inhibit furin-like and SARS-CoV-2 infection activity [80]. In a mouse model of pneumonia, treatment with polyphenol-rich extract from *M. officinalis* bark lowered high levels of NO, IL-6 and TNF-α expression and provided long-term protection against reinfection [81, 82].
10. Gualou (*Trichosanthes kirilowii Maxim.*)
  *T. kirilowii* tuber extract has long been used in Eastern Asian medicine to alleviate diabetes symptoms. Cucurbitacin D has been isolated from *T. kirilowii* and has shown anti-inflammatory and anticancer activities. Cucurbitacin D induces apoptosis and suppresses tumor cell proliferation by inhibiting signal transducer and activator of transcription 3 (STAT3) and phosphorylating NF-κB [83, 84]. Trichosanhemiketal A and B from *T. kirilowii* inhibit iNOS and COX-2 expression [85]. *T. kirilowii* trichosanthin extract inhibits HIV-1 replication and displays antitumor activity [86].
11. Yinchen* (Artemisia scoparia)*
  *A. scoparia* is used for hepatoprotective, choleretic, and diuretic effects. Scientific evidence suggests wide-ranging in vivo and in vitro biological activity of *A. scoparia* extracts and active constituents, covering anticancer, anti-inflammatory, antibacterial, hepatoprotective, anti-atherosclerotic, antiviral and neuroprotective functions. *A. scoparia* total flavonoids suppress the production of NO, ROS, TNF-α, IL-6, MCP-1 and prostaglandin E2 (PGE2) and strongly inhibit the degradation of nuclear factor kappa B α (IκBα) as well as the nucleus translocations of phosphoryl-p65, p-p38 and phosphate- extracellular signal regulated kinase 1/2 (p-ERK) in RAW264.7 cells after LPS stimulation [87]. *A. scoparia* total flavonoids also decrease the lung wet: dry weight ratio in lung tissue specimens from mice with acute lung injury [87]. *A. scoparia* water extract has potent anti-inflammatory activity in macrophages and alleviates carrageenan-induced acute inflammation in mice [88]. Scopolein, a coumarin isolated from *A. scoparia*, suppresses the production of proinflammatory cytokines and inhibits LPS-induced PGE2 production by reducing COX-2 expression [89]. Scopoletin also ameliorates synovial inflammation and lessens the destruction of cartilage and bone in rats with adjuvant arthritis by reducing IL-6 expression and inhibiting the phosphorylation of p38 mitogen-activated protein kinase, ERK, and protein kinase C (PKC) [90]. The *A. scoparia* flavonoid cirsimaritin can reduce influenza virus A (IAV) viral titers and protein synthesis by inactivating the NF-κB/p65 signaling pathway [91]. Around 90% of *A. scoparia* ethanol extracts exhibit anti-hepatitis B virus (HBV) activity [92], while other important compounds isolated from *A. scoparia* exhibit inhibitory effects on coronoviruses. Notably, another flavonoid compound isolated from the *Artemisia* genus, isorhamnetin, inhibits the activity of SARS-CoV-2 spike pseudotyped virus via the binding of ACE2, indicating that isorhamnetin may have therapeutic potential against COVID-19 [93].
12. Fuling* (Wolfiporia extensa)*
  *Wolfiporia extensa* is an edible medicinal mushroom that is used in the treatment of edema, insomnia, spleen deficiency, vomiting, and other diseases. Edible mushrooms have recently been used as functional foods or dietary supplements. Pharmacological effects of W. *extensa* include antitumor, antioxidant, anti-inflammatory and antibacterial activities, immunomodulatory effects and immune enhancement. Polysaccharides have strong antitumor and hepatoprotective effects. Polysaccharides and triterpenes are the main biologically active compounds of *W. extensa* [94]*.* Pachymic acid and dehydrotumulosic acid isolates from *W. extensa* have inhibited acute ear edema, which indicates considerable anti-inflammatory potency [95]. A 50% hot ethanol extract from *W. extensa* dose-dependently increased the in vitro secretion of IL-1β and IL-6 in human peripheral blood monocytes [96], while Fuling polysaccharide has stimulated RAW264.7 macrophages via the induction of TNF-α and IL-1β and the regulation of NF-κB-related gene expression [97].
13. Yuzhu* (Polygonatum odoratum)*
  *P. odoratum* has shown strong clinical efficacy in the treatment of diabetes, microbial infections, inflammation, and tumors. *P. odoratum* and its main constituents scutellarein-7-glucoside and quercitrin are associated with reduced IL-6 secretion in LPS-stimulated macrophages [98]. Three anthraquinone analogues have been isolated in phytochemical research involving ethyl acetate-soluble ingredients extracted from the roots of* P. odoratum* [99]*.* Polysaccharides from *P. odoratum* promote the proliferation and neutral red phagocytosis of RAW 264.7 macrophages [100].
14. Banxia *(Pinellia ternata)*
  *P. ternata* is extremely effective in the treatment of cough, vomiting, infection, and inflammatory diseases. *P. ternata* contains many alkaloids, iridoids, iridoid glycosides, anthraquinones, anthraquinone glycosides, fatty acids and their derivatives. Pharmacologic investigations have shown that *P. ternata* has antidepressant, wound healing, antitussive, antiemetic, antifungal, anti-inflammatory, sedative-hypnotic, antioxidant and insecticidal activities [101]. *P. ternata* also inhibits cancer cell proliferation and convulsive seizures [101]. In rats with chronic obstructive pulmonary disease, *P. ternata* treatment reversed the increases in IL-1β and TNF-α levels in bronchoalveolar lavage fluid (BALF) after budesonide withdrawal and protected the airway from mucus hypersecretion and airway inflammation after inhaled corticosteroid withdrawal [102].
  In a mouse model of allergic asthma, *P. ternata* water extract significantly attenuated the ovalbumin (OVA)-induced influx into the lungs of total leukocytes, eosinophils, neutrophils, macrophages and lymphocytes, and dose-dependently reduced the levels of IL-4, IL-13 and TNF-α [103]. *P. ternata* water extract also significantly reduced the plasma levels of total and OVA-specific immunoglobulin (Ig)E release into the airspace and inhibited OVA-induced increases in MUC5AC (mucin 5AC, oligomeric mucus/gel-forming) and iNOS expression [103].
15. Fuzi* (Aconitum carmichaelii)*
  For more than 2000 years, Fuzi (the lateral root of* A. carmichaeli)* has been used for its anti-inflammatory, analgesic and cardiotonic effects, and is mainly used in TCM to treat musculoskeletal disorders. Fuzi contains the highly toxic C19 diterpenoid alkaloids aconitine, mesaconitine and hypaconitine. Fuzi ingestion can result in typical signs and symptoms of aconitine poisoning. Ventricular arrhythmias can lead to death, most likely within the first 24 h. Cases of poisoning are still reported in areas where herbal medicine is used, including China, Taiwan, and India [104]. Using these toxic drugs may sound dangerous. However, *A. carmichaeli* has an important role in many diseases, including rheumatism, arthralgia, edema, gastroenteritis, asthma, abdominal pain, and some gynecological diseases such as irregular menstruation and dysmenorrhea [105]. Moreover, *A. carmichaeli* is a very effective pain reliever herb due to its action upon neuronal cells [105]. Therefore, it is important to study its effects, toxicity and therapeutic relevance in depth. The processed product of *A. carmichaeli* reduces serum TNF-α and IL-β levels in the rat adjuvant arthritis model [106]. Crude Fuzi combined with glycyrrhiza reduces the risk of heart failure by ameliorating the inflammatory response, which researchers suspect could be partly related to the inhibition of the Toll-like receptor-4 (TLR4)/NF-κB action in the heart [107].

**Table 4:** The potential efficacy of the herbs of NRICM101 and NRICM102 used as adjunctive therapies in COVID-19 infection

Ingredients	Models	Active ingredients	Mechanisms of action	References
*Morus alba* L	Vero and HeLa cells	Kuwanon X	Anti-HSV-1, HSV-2 and reduced the expression of NF-κB	[43, 44]
	MDCK cells	Aqueous extracts	Exhibited H1N1 and H3N2 inhibitory activity	[48]
	Molecular docking		Anti-TMPRSS2 activity	[49]
	RAW264.7 cell and animal model	30%, 50%, and 75% ethanol extract	Reduced the production of pro-inflammatory cytokines and decreased expression of NO, iNOS and COX-2	[50]
*Saposhnikovia divaricata* (Turcz. ex Ledeb.) Schischk	Vero cells	Methanol extract	Inhibited the PEDV inhibitor	[48, 49]
	RAW 264.7 cells	70% ethanol extract	Inhibited the production of NO, PGE2, TNF-α, and IL-6	[50]
*Isatis tinctoria* subsp. tinctoria	Vero cells	Indigo, sinigrin, aloe-emodin, and hesperetin	Anti-SARS coronavirus 3C-like protease effects	[52]
	HepG2	Lariciresinol	Inhibited hepatitis B virus	[53]
	Human epidermal keratinocytes and mice	Petroleum ether extract	Inhibition of IL-6, IL-33 and mast cell degranulation	[54]
*Mentha canadensis* L	VeroE6/TMPRSS2 cells	Aqueous extracts	Inhibited SARS-CoV-2 virus infection	[56]
	RAW264.7 cells, human epidermal keratinocyte and mice	Essential oil	Inhibited the expression of NO, TNF-α, IL-1β, and IL-6 and reduced the expression of ERK/NF-κB signaling pathways	[57]
*Nepeta tenuifolia* Benth	HG23 cells	80% methanol extract	Inhibited norovirus replication through the induction of antiviral interferon production during virus replication	[59]
	Vero cells	Aqueous extract	Inhibited the synthesis of viral RNA and protein	[60]
	Peritoneal macrophages and mice	Aqueous extract	Inhibited the expression of TNF-α and IL-6 and reduced the expression of IκB-α degradation and activation of c-Jun and ATF-2	[61]
*Scutellaria baicalensis* Georgi	C6/36 mosquito cell line	Aqueous extract	Antiviral activities in ZIKA22, H1N123, HIV24, and DENV25 infections	[62, 63]
	Vero cells	Baicalin, baicalein wogonin	Inhibits replication of SARS-CoV-2 and its 3C-like protease	[64]
	RAW264.7 cell & animal model	Aqueous extract	Reduced iNOS, COX-2, TNF-α and IL‑1β levels, and NF-κB, MAPK phosphorylation	[65]
*Houttuynia cordata* Thunb	HeLa 229 cells	Aqueous extract	Blocked herpes simplex virus (HSV) infection via inhibition of NF-κB activation	[67, 68]
	Mouse peritoneal macrophages & animal model	Volatile oil	Suppressed NO, iNOS and TNF-α	[69]
	Mouse liver epithelial cell line	Quercetin, quercetrin, cinanserin	Antiviral activities in murine coronavirus and dengue virus infections	[70–72]
*Glycyrrhiza uralensis* Fisch. ex DC	Huh7.5 cells	Methanol extract, glycycoumarin, glycyrin, glycyrol and liquiritigenin isolated, isoliquiritigenin, licochalcone A and glabridin	Anti-hepatitis C virus	[74]
	Vero cells	Glycyrrhizin	Anti-SARS virus	[75, 76]
	RAW264.7 cells	Glycyrrhizin and soliquiritigenin	Suppressed IL-6 and TNF-α production	[77]
*Magnolia officinalis* Rehder & E.H.Wilson	Vero cells	*H. officinalis* extract, Honokiol	Anti-human norovirus surrogates, MNV and FCV	[79]
	A549 cells	Honokiol	Blocked furin-like activity and SARS-CoV-2 virus	[80]
	RAW264.7 cells	Magnolol	Inhibited the expression of TNF-α, IL-6 and IL-1β, reduced the expression of the NF-κB, TLR4 and MAPK pathways	[81]
	Mice	Polyphenol-rich extract	Reduced serum levels of NO, IL-6 and TNF-α in influenza virus-induced pneumonia	[82]
*Trichosanthes kirilowii* Maxim	MCF7, SK-BR3, and MDA-MB-231 breast cancer cells	*Trichosanthes kirilowii* ethanol extract and cucurbitacin D	Bocked tumor cell proliferation and induced apoptosis	[83, 84]
	RAW264.7 cells	Trichosanhemiketal A and B	Suppressed NO, iNOS and COX-2	[85]
	H9 and C8166 cells	Trichobitacin	Anti-HIV-1 activity	[86]
*Artemisia scoparia* Waldst. et Kit	BMDMs, 3T3-L1, THP-1 cells and C57BL/6 mice	Aqueous extract	Reduced production of the inflammatory cytokines IL-6, TNF-α, CXCL1, and IL-1β	[87, 88]
	RAW264.7 cells	Scopolein	Suppresses the production of proinflammatory cytokines and inhibits LPS-induced PGE2 production through the depression of COX-2 expression	[89, 90]
	MDCK and THP-1 cells	Cirsimaritin	Inhibited IAV replication by downregulating the NF-κB signaling pathway	[91]
	HepG2	ethanol extracts	Anti-hepatitis C virus	[92]
	ACE2^h^ cells	isorhamnetin	Anti-SARS virus via the binding of ACE2	[93]
*Wolfiporia extensa* (Peck) Ginns	RAW264.7 cells and C57BL/10ScNJ mice	Polysaccharide	Increased nitric oxide, IL-2, IL-6, IL-17 A, TNF, and IFN-γ in macrophages and reduced tumor volume	[94, 97]
	BALB/c mice	Pachymic acid and dehydrotumulosic acid	Acute ear edema, indicating considerable anti-inflammatory potency	[95]
	Human peripheral blood monocytes	50% hot ethanol extract	Increased the secretion of IL-1β and IL-6	[96]
*Polygonatum odoratum* (Mill.) Druce	RAW264.7 cells	Scutellarein-7-glucoside; quercitrin	Reduced IL-6 secretion in LPS-stimulated macrophages	[98]
	293 T/MDCK cells	Athraquinone analogues	Exhibited H1N1 inhibitory activity	[99]
	RAW264.7 cells	Polysaccharides	Promoted in vitro proliferation and neutral red phagocytosis of RAW 264.7 macrophage cells	[100]
*Pinellia ternata* (Thunb.) Makino	SD rats	*P. ternata* (crude herb)	Anti-coughing effects, by attenuating the budesonide withdrawal-induced rebound in goblet cell numbers, MUC5AC expression, IL-1β and TNF-α release, and ERK activity	[101, 102]
	BALB/c mice	Aqueous extract	Attenuated the OVA-induced increase in IL-4, IL-13, TNF-α, mucin 5AC and iNOS expression	[103]
*Aconitum carmichaeli* Debeaux	SD rats	Aqueous extract (processed *A. carmichaeli*)	Reduces serum TNF-α and IL-β levels in the rat adjuvant arthritis model	[106]
	C57BL/6 J mice	Aqueous extract	Protects against ischemia-induced heart damage by attenuating inflammatory responses through the TLR4/NF-κB pathway	[107]

### TCM for clinical management of COVID-19 in Taiwan

Currently, four TCM formulations intended for use against COVID-19 have been subjected to clinical research in Taiwan and the published data are providing policymakers and healthcare personnel with the opportunity to prepare for future epidemics in Taiwan (Table 1).

### Clinical efficacy of Jing Si Herbal Tea in Taiwan

In this study, 260 patients with mild-to-moderate COVID-19 infection were allocated to the JSHT group (n = 117) or control group (n = 143), in combination with standard management. JSHT therapy was associated with reductions in SARS-CoV-2 viral load, systemic inflammatory markers (IL-6, IL-8, and IL-10), and lung infiltrates, especially in male and elderly patients (aged ≥ 60 years). Mortality rates were 0.0% in the JSHT group and 2.8% in the control group, indicating that JSHT combined with standard management may prevent the development of critical illness and mortality in patients with mild-to-moderate COVID-19 [39, 40].

### Clinical efficacy of Jing Guan Fang in Taiwan

Responses have been analyzed from 396 Taiwanese patients (mean age 45.9 years; 35.1% male, 64.9% female) with COVID-like symptoms who completed a questionnaire about their experiences of JGF administered as a complementary preventative strategy. Sore throat was the most common COVID-19-like symptom, affecting 34 patients; at 7 days after consuming JGF, 31 (91.2%) of those patients reported an improvement in symptoms. Similarly, among patients who reported non-COVID-19-like symptoms of fatigue (n = 128) and nervousness (n = 102), the majority reported improvements in these symptoms at 7 days after taking JGF (81.3% and 68.6%, respectively) [41].

### Clinical efficacy of NRICM101/102 in Taiwan

Data have been analyzed from 840 patients with COVID-19 infection admitted to 9 hospitals in Taiwan during 2021. The 2 study cohorts consisted of 302 patients (151 received NRICM101 and 151 did not) and 246 patients (123 received NRICM102 and 123 did not). During the 30-day observation period, patients who received NRICM101 plus usual care had no end point events, while 14 (9.27%) in the usual care group were intubated or admitted to the intensive care unit (ICU). Seven deaths (5.69%) occurred in the NRICM102 plus usual care group compared with 27 (21.95%) in the usual care group. Patients who did not receive NRICM101 transitioned to more severe states; NRICM102 users were 74.07% less likely to die than non-users. The data support the therapeutic efficacy of NRICM101 and NRICM102 in addition to usual care. NRICM101 and NRICM102 represent therapeutics with broad-spectrum active compounds that inhibit host cell pathways necessary for viral infection and replication and help to maintain bodily function to counter the effects of viral diseases. Both formulations have inhibitory effects on the five main variants of COVID-19 (Alpha, Beta, Gamma, Delta and Omicron), which addresses concerns about the evolution of the virus. This study provides important information about the adoption of oral therapy and evidence in support of NRICM101 and NRICM102 shortening response times to epidemic outbreaks, offering the potential for novel, safe treatments for future epidemics [13, 14].

### Theoretical perspectives on herb-herb combinations

Many theories around the therapeutic concepts of TCM draw upon philosophical ideas such as the balance of yin and yang [108]. TCM seeks to establish that diseases develop in response to imbalances in various parts of the body, and that the use of herbal remedies and other therapeutic modalities, such as acupuncture and physical manipulations, can restore a state of balance. A set of TCM systems guides the clinician’s selection of medicines for the treatment of diseases [109]. Most TCM prescriptions contain more than one medicine, forming a multi-item mixture (compound). When prescribing medicines, clinicians must consider the principles of interaction between herbs in order to formulate the best prescription for therapeutic effect [110]. Combination effects, whether complementary or antagonistic, will be reflected in clinical outcomes [111, 112].

The compatibility theory of Chinese herbal medicine endorses the combination of more than one drug, according to the needs of the disease. The compatibility theory provides Chinese medicine with guiding principles for improving the safe and effective use of herbal medicines. This theory was first recorded in “Shen Nong’s Materia Medica”, a classic work that describes TCM [113]. From a modern pharmacologic point of view, this is a theory of herbal interactions. A total of seven possible outcomes refer to a single effect: mutual accentuation; mutual enhancement; mutual counteraction; mutual suppression; mutual antagonism; and mutual contrariness; all of which describe the interactions between drugs [114, 115].

“Eighteen Antagonisms” are representatives of incompatibility in clinical records of TCM. This is a special regulation for the compatibility of traditional Chinese medicine prescriptions, because mutual antagonism can lead to adverse reactions. Clinicians are most concerned with “contradictory” reactions, as this may involve clinical “adverse drug reactions”. Most Chinese medicine practitioners endorse the “eighteen antis” as a clinically recognized taboo, which dictates that a total of 18 substances across three groups of medicines should be avoided in combination to prevent increases in toxic and side effects [113]. However, famous physicians in ancient times often issued prescriptions that violated the eighteen antis [114, 116]. For example, Zhang Zhongjing’s “Essentials of the Golden Chamber” included “Gansui Banxia Decoction”, which combines gansui and licorice [117, 118]. Some medical experts also believe that medicinal use of mutually contrary drugs can complement each other and produce strong effects. If used properly, such medicinal use can cure some clinical conditions. Thus, some clinicians believe that clinical medicine should not be limited to the “eighteen antagonisms”.

### Pharmacological research on the synergism among *Aconitum carmichaeli, Trichosanthes kirilowii,* and *Pinellia ternate*

The mixed use of *A. carmichaeli*, *T. kirilowii* and *P. ternate* in NRICM102 violates the TCM compatibility (“eighteen antagonisms”) principle and may produce or enhance toxic reactions or side effects [15] **(**Table 5**)**.

1. The compatibility of* A. carmichaeli* and *T. kirilowii*
  The “eighteen antagonisms” specifies that *A. carmichaeli* and *T. kirilowii* should not be used together, although scant evidence supports this dictum. The main functions of aconite are to restore and assist yang in relieving inferiority, dispel cold to relieve pain, and to treat diseases such as heart failure and wind-cold-dampness arthralgia. The therapeutic effects of *T. kirilowii* include the clearing away of heat and expectoration of phlegm, widening of the chest and dispelling of knots, moisturizing dryness and smoothing the intestines [119]. Although *A. carmichaeli* and *T. kirilowii* are considered anti-drugs and there are contraindications for compatibility, some historical records indicate that the two can be used together for treatment of disease. There are also examples of modern medicine using these substances in combination. *A. carmichaeli* and *T. kirilowii* have been commonly used in TCM for the treatment of heart disease since the time of Zhang Zhongjing during the Han Dynasty. From the perspective of TCM for the treatment of heart failure, aconite and cinnamon sticks are appropriate for patients with yang deficiency, while melon wilt and the like are added for patients with yang deficiency and phlegm turbidity. This method is consistent with the TCM theory of warming yang and removing turbidity [120]. In rats with chronic pressure-overload heart failure in the early stage, the combination of the dried tuber of *A. carmichaeli* and *T. kirilowii* appears to be beneficial [121], whereas the same therapeutic combination appears to aggravate heart and kidney inflammation and promote myocardial fibrosis in rats with middle- or late-stage chronic heart failure [122]. More research is needed to confirm this.
2. The compatibility of *A. carmichaeli* and *Pinellia ternate*
  A review of the ancient and modern literature, combined with an investigation of clinical prescriptions, reveals a large body of evidence showing that the combination of Fuzi and *Pinellia* in the treatment of heart failure, chest tightness and pain synergistically enhances efficacy and reduces toxicity. In these therapeutic indications, the function of TCM is to restore yang and relieve inversion, tonify fire and assist yang, and eliminate wind, cold and dampness. Preprocessed aconite contains a large amount of aconitine, which will cause cardiac paralysis that in turn causes death; processed aconite is less toxic. Fuzi, one of the oldest and most commonly used TCM substances for treating heart failure, exhibits many pharmacological effects, including cardiotonic, anti-inflammatory, analgesic and antitumor activities, and hemodynamic improvement [123]. The combination of aconite and *Pinellia* first appeared in the “Golden Chamber Synopsis”, in which the Fuzi Japonica Decoction was prescribed by Zhang Zhongjing to treat the symptoms of thunder and pain in the abdomen, chest and flank fullness and vomiting in the upper offenders of the internal resistance of yin, cold and dampness. This decoction consists of Pao Fuzi, *Pinellia*, licorice, jujube, and japonica. In the prescription, aconite tonifies yang and benefits fire, dispels cold and relieves pain. When yang is prosperous, the evil of yin cold and damp turbidity will be naturally eliminated. Sun Simiao’s “Qianjin Yaofang” from the Tang Dynasty records the use of Dawuyin Pills, while the official medical book “Taiping Huimin Mixture Jufang” from the Song Dynasty describes the use of the Shishiwei Jianzhong Decoction with aconite *Pinellia*. Thus, Fuzi and *Pinellia* in combination has potential for the treatment of various diseases.
  A statistical analysis of the “Chinese-Italian Dictionary of Prescriptions” records 502 classic prescriptions using both Fuzi and *Pinellia*. This combination can reduce the toxicity and improve the pharmacological effects of each substance. In animal models of chronic heart failure, Fuzi compatibility with other reviewed study treatments was superior to Fuzi alone for the treatment of chest tightness and pain and was associated with reduced toxicity [123].
  Pharmacologic research has found that the combination of aconite and *Pinellia* inhibits the β2-adrenergic receptor / stimulatory G protein / protein kinase A (β2-AR/Gs/PKA) signal, which may explain the anti-inflammatory effects, antifibrosis and improvement of cardiac function that occurs with this therapy, although other data have described how aconite combined with *Pinellia* aggravates doxorubicin-induced cardiomyopathy associated with the β2-AR activation of Gs, the stimulatory G protein for adenylyl cyclase [124]. It is important to recognize the existence of antagonistic pharmacologic effects within a single TCM substance that can result in different or opposite effects. For instance, the four compounds in aconite (talatizamine, 14-acetyl-latizamin, hetisine, and 14-benzoylnovine) can counteract doxyrubicin-induced heart failure [125], while aconitine in aconite can induce apoptosis in H9c2 cardiomyocytes [126]. Aconite also produces antagonistic chemical components that play a role in antiarrhythmic effects, such as N-aconitine, N-deacetylaconitrile, raconitine, aconitine, 14-acetyl taladiamine, and aconitine [127]. Thus, different active ingredient composition ratios of aconite and *Pinellia* may induce different or antagonistic effects from those originally intended.
3. The Compatibility of *A. carmichaeli and Ampelopsis japonica*
  A formula combining *A. carmichaeli* with *A. japonica* was first recorded in “Da Ba Feng San” by Sun Simiao (Tang Dynasty) in his “Qian Jin Fang” [128]. The TCM fumigation and washing method for the formula containing *A. carmichaeli* and *A. japonica* produces a significant curative effect in knee osteoarthritis, with a very high cure rate of 96.55% [129]. When arthritis is treated with these two herbs and others in combination with sodium hyaluronate, joint swelling has been eliminated, with accompanying pain relief [130]. *A. japonica* has therapeutic potential in the treatment of rheumatoid arthritis [131].
  The use of “eighteen antagonisms” drugs in prescriptions that may cause or aggravate known side effects is controversial and has prevented the clinical application of many TCM herbal combinations [119]. However, NRICM102 treatment of patients with COVID-19 has been associated with greatly reduced mortality rates compared with conventional medical treatment and no toxic reactions have been recorded with NRICM102; 1 patient death has occurred due to severe COVID-19 infection [15]. Moreover, 30-day toxicity test results from mice administered NRICM102 revealed no abnormal tissue or organ reactions [15].
  From the treatment perspective, patients with severe pneumonia complicated by cardiopulmonary failure who need to use aconite to enhance cardiopulmonary function usually also have pulmonary edema and hydropleural effusion and require *Pinellia* and *Trichosanthes* to clear the respiratory tract [100, 101]. Such patients have sputum in the pleural cavity, which is unlikely to be due to the toxic drug combination that ancient physicians believed to be contributing to the patient’s rapid clinical deterioration and death. Those physicians believed that (1) it is too late to reverse the disease pathogenesis and prevent rapid deterioration and (2) strong aconitine toxicity exists due to incomplete processing of aconitum. We believe that rigorous drug research and development, combined with diagnoses and treatments prescribed by professional and experienced clinicians, will support the combination of Fuzi, *Pinellia* and *Trichosanthes* for improved clinical efficacy and rescue patients in a timely manner [83]. Combinations of TCM substances and interactions between Chinese and Western medicine are important clinical issues that deserve full attention.

**Table 5:** Traditional knowledge of aconite unpaired in “eighteen antagonisms”

Ingredient	Antagonistic medicaments	Mechanisms	References
*Aconitum carmichaeli*	*Trichosanthes kirilowii*	Aggravated inflammatory responses in the heart and kidney and promoted myocardial fibrosis by activating β2-AR/PKA signaling in rats with chronic pressure-overload heart failure	[121]
		Improves hemodynamics and exerts myocardial protection in rats with chronic heart failure	[122]
	*Pinellia ternata*	Fuzi and Pinellia combination aggravated doxorubicin-induced cardiomyopathy associated with PKA/β2AR-Gs signaling	[125]
		The PKA/β2-AR-Gs/Gi signaling pathway is associated with anti-inflammatory and proapoptotic effects of the Fuzi and Pinellia combination in rats with pressure-overload heart failure	[126, 127]
	*Ampelopsis japonica* root	*Aconitum carmichaeli* combined with *Ampelopsis japonica* has superior therapeutic effects in rheumatoid arthritis compared with either treatment alone	[130, 131]
	*Bletilla striata* rhizome	–	–
	*Fritillaria* spp. bulb	–	–

## Conclusion

Although the Omicron mutant strain has greatly reduced the toxicity and lethality of COVID-19 infection, the COVID-19 epidemic continues to cause anxiety. For infected patients isolated at home, TCM can be used to prevent severe illness. Scientific evidence shows that TCM ingredients clear away heat and detoxify, remove moisture from the body’s surface and inhibit viruses. TCM can improve the symptoms of COVID-19, slow clinical deterioration, and reduce all-cause mortality in severe COVID-19 infection. The therapeutic efficacy of TCM may be improved by using different combinations of compounds. TCM antiviral activity occurs either through the direct inhibition of the entry and replication of the virus, with the use of substances such as *Houttuynia* and *Scutellaria* to clear heat and detoxify [132], or by indirectly exerting antiviral effects or inhibiting virus-mediated inflammatory responses by regulating the immune function of organisms. Licorice and mint are used as antivirals and induce interferon and immunoglobulin [10]. As TCM focuses on the overall regulation of the body, such therapy can play a multitargeted role in the regulation of body systems [133, 134]. Effecting a cure of a complex disease such as COVID-19 may not be possible with a single targeted drug, whereas the multicomponent and multitargeted nature of Chinese medicine suggests that such treatment has good potential to supplement standard medical treatment in COVID-19 [135, 136]. An important aspect of different Chinese medicines is that they may share the same or similar targets and pathways. Improving our understanding of how underlying mechanisms of action of TCM ameliorate symptoms of COVID-19 will help us to improve the use of TCM in the preventing and treating COVID-19 infection, help us to understand how molecular mechanisms associated with TCM can improve organ damage caused by COVID-19, and use modern scientific techniques to determine the material basis of possible drugs. This review is intended as a reference for the development of new drugs for preventing and treating viral infections such as COVID-19. NRICM102 is an herbal formula that has been developed according to TCM theory and preclinical experimental data. Mechanism of action studies reveal that the therapeutic effects of NRICM102 occur through the comprehensive regulation of multiple targets and multichannel synergistic mechanisms. While the global COVID-19 outbreak shows no sign of lessening, NRICM102 may be used in addition to conventional drug therapy for treating severe cases of infection.

## Data Availability

Not applicable.

## Abbreviations

COVID-19: Coronavirus disease 2019
SARS-CoV-2: Severe acute respiratory syndrome coronavirus 2
CAMs: Complementary and alternative medicines
WHO: World Health Organization
TCM: Traditional Chinese medicine
VOC: Variants of concern
mAbs: Monoclonal antibodies
RdRp: RNA-dependent RNA polymerase
3CL^pro^: 3C-like protease
M^pro^: Main protease
FDA: Food and drugs administration
EUA: Emergency use authorization
NHC: National health commission
CFDA: China cood and drug administration
AYUSH: Ayurveda, yoga and naturopathy, unani, siddha and homeopathy
JSHD: Jing Si herbal drink
ACE2: Angiotensin converting enzyme-2
TMPRSS2: Transmembrane protease, serine 2
SOD: Superoxide dismutase
iNOS: Inducible nitric oxide synthase
NF-κB: Nuclear factor-κB
NO: Nitric oxide
TNF-α: Tumor necrosis factor-α
IL-6: Interleukin 6
JGF: Jing Guan Fang
NRICM: National research institute of Chinese medicine
NRICM101: Taiwan Chingguan Yihau
NRICM102: Taiwan Chingguan Erhau
H1N1: Influenza A
HIV: Human immunodeficiency virus
DENV: Dengue virus
MCP-1: Monocyte chemoattractant protein-1
CXCL-10: Interferon-inducible protein-10
VEGF: Vascular endothelial growth factor
LPS: Lipopolysaccharide
HSV-1: Herpes simplex virus 1
COX-2: Cyclooxygenase-2
HCV: Hepatitis C virus
MNV: Murine norovirus
FCV: Feline calicivirus
STAT3: Signal transducer and activator of transcription 3
PGE2: Prostaglandin E2
IκBα: Nuclear factor kappa B α
p-ERK: Phosphate- extracellular signal regulated kinase 1/2
ERK: Extracellular signal regulated kinase
PKC: Protein kinase C
IAV: Influenza virus A
BALF: Bronchoalveolar lavage fluid
OVA: Ovalbumin
MUC5AC: Mucin 5AC, oligomeric mucus/gel-forming
TLR4: Toll-like receptor-4
ICU: Intensive care unit
β2-AR/Gs/PKA signal: β2-Adrenergic receptor/stimulatory G protein/protein kinase A signal

## Footnotes

1 Palpation is usually performed by a healthcare practitioner and refers to the process of using one's hands to feel an object in or on the body to determine its size, shape, firmness, or location, especially while perceiving/diagnosing a disease or illness. Pulse-taking is another TCM method of disease diagnosis, whereby a TCM practitioner touches the pulse in different parts of the human body and observes the changes in pulse conditions. This technique is also known as cutting pulse, diagnosing pulse, pressing pulse, or holding pulse.
